# Developmental Delay and Male-Biased Sex Ratio in *esr2b* Knockout Zebrafish

**DOI:** 10.3390/genes15050636

**Published:** 2024-05-17

**Authors:** Wei Peng, Yunsheng Zhang, Bolan Song, Pinhong Yang, Liangguo Liu

**Affiliations:** 1 College of Life and Environmental Sciences, Hunan University of Arts and Science, Changde 415000, China; yskaoyan@163.com (Y.Z.); songbolantop1@huas.edu.cn (B.S.); yph588@163.com (P.Y.); llg1818@126.com (L.L.); 2 State Key Laboratory of Development Biology of Freshwater Fish Sub-Center for Health Aquaculture, Changde 415000, China

**Keywords:** zebrafish, gene knockout, *esr2b*, sex ratio

## Abstract

The estrogen receptor signaling pathway plays an important role in vertebrate embryonic development and sexual differentiation. There are four major estrogen receptors in zebrafish: *esr1*, *esr2a*, *esr2b* and *gper*. However, the specific role of different estrogen receptors in zebrafish is not clear. To investigate the role of *esr2b* in zebrafish development and reproduction, this study utilized TALENs technology to generate an *esr2b* knockout homozygous zebrafish line. The number of eggs laid by *esr2b* knockout female zebrafish did not differ significantly from that of wild zebrafish. The embryonic development process of wild-type and *esr2b* knockout zebrafish was observed, revealing a significant developmental delay in the *esr2b* knockout zebrafish. Additionally, mortality rates were significantly higher in *esr2b* knockout zebrafish than in their wild-type counterparts at 24 hpf. The reciprocal cross experiment between *esr2b* knockout zebrafish and wild-type zebrafish revealed that the absence of *esr2b* resulted in a decline in the quality of zebrafish oocytes, while having no impact on sperm cells. The knockout of *esr2b* also led to an abnormal sex ratio in the adult zebrafish population, with a female-to-male ratio of approximately 1:7. The quantitative PCR (qPCR) and in situ hybridization results demonstrated a significant downregulation of *cyp19ab1b* expression in *esr2b* knockout embryos compared to wild-type embryos throughout development (at 2 dpf, 3 dpf and 4 dpf). Additionally, the estrogen-mediated induction expression of *cyp19ab1b* was attenuated, while the estradiol-induced upregulated expression of *vtg1* was disrupted. These results suggest that *esr2b* is involved in regulating zebrafish oocyte development and sex differentiation.

## 1. Introduction

The production of estrogen is mediated by P450 cytochrome enzymes, which facilitate the synthesis of testosterone and play a pivotal role in various physiological processes such as reproduction and development in vertebrates. In addition to reproductive regulation, estrogen affects the development and maintenance of numerous organs such as the bones, cardiovascular system and nervous system [1]. The primary process of the estrogen receptor signaling pathway involves the binding of estrogen to its receptor, which subsequently recognizes and binds to the estrogen response element on the target gene to regulate gene expression, thereby achieving physiological function regulation [2].Currently, mammals possess two estrogen receptors (ERα and ERβ) that are widely distributed throughout the hypothalamic–pituitary–gonadal reproductive axis, encompassing both male and female individuals [3,4]. The knockout of the estrogen receptor has been conducted in mice from as early as the 1990s; ERα knockout homozygous mice were found to be infertile, with subsequent examination revealing uterine hypoplasia and the absence of the corpus luteum in ovaries [5,6,7,8]. The knockout of mouse ERα also led to a significant elevation in estrogen and LH levels in the bloodstream, potentially attributed to the disruption of estrogen’s feedback regulation caused by Erα knockout [9,10]. Knockout of ERβ in mice results in decreased reproductive ability, but gonadotropin expression remains unaffected, indicating that ERβ is not involved in estrogen feedback regulation [6,11,12,13].

Zebrafish possess four primary estrogen receptors—namely, *esr1*, *esr2a*, *esr2b* and *gper*. Among these receptors, *esr1* corresponds to ERα in mammals while *esr2a* and *esr2b* correspond to ERβ2 and ERβ1, respectively [14,15,16]. *Esr1*, *esr2a* and *esr2b* all belong to nuclear receptors, whereas *gper* belongs to membrane receptors and lacks a DNA-binding domain [17]. The findings of various studies have demonstrated that *gper* has the ability to bind with 17β-estrogen and initiate downstream G protein signaling pathways in order to carry out its biological functions [17]. The expression of *esr2a* mRNA in egg cells occurs at high levels during early embryonic development, gradually declining at 8 hpf. Prior to 12 hpf, the expression of *esr1* and *esr2b* has been observed to be minimal [18,19]. However, from 12hpf onwards, there is a significant increase in the expression of *esr1*, while for *esr2b*, this increase occurrs from 24 hpf [18,19]. The MO injection technique is a widely used approach for investigating gene function during the early stages of embryonic development in zebrafish. The injection of *esr2a* MO by Froehlicher into transgenic zebrafish embryos with hair cell fluorescent markers resulted in a significant reduction in fluorescence signal intensity [20]. Additionally, the results of in situ hybridization have demonstrated that *esr2a* injection leads to a notable decrease in the number of hair cells, indicating the involvement of *esr2a* in early sensory neuron development [20]. A study conducted by Celeghin demonstrated that the administration of *esr2a* MO can induce apoptosis in brain cells within zebrafish embryos, consequently resulting in developmental abnormalities encompassing delayed development, body curvature and cerebral malformations [21]. Another study on *esr1* demonstrated that the manipulation of *esr1* expression levels in early zebrafish embryos results in aberrant cell migration [22]. To investigate the role of estrogen receptors in reproduction, Lu et al. used CRISPR/Cas9 technology to construct *esr1*, *esr2a* and *esr2b* knockout zebrafish [23]. Surprisingly, a comparative examination of gonadal development in adult zebrafish lacking the estrogen receptor (ER) revealed no apparent abnormalities, except for an atypical female-to-male ratio, characterized by a significantly higher proportion of males within the population [23].

*Cyp19a1b* and *vtg1* are typical estrogen response genes that are used as biomarkers of estrogenic endocrine-disrupting pollution [24,25,26]. In zebrafish, there exist two cytochrome P450 enzymes, encoded by *cyp19a1a* and *cyp19a1b* [27,28]. *Cyp19a1a* is not an estrogen response gene and is mainly expressed in the zebrafish ovary [29,30]. *Cyp19a1b*, the brain-specific aromatase B, is expressed mainly in radial glial cells of the brain [31]. *Cyp19a1b* mRNA is detected in unfertilized eggs, disappears at 12 hpf and recovers with the start of the zygotic expression [32]. Then, the *cyp19a1b* mRNA level strongly increases from 24 hpf to 48 hpf [32]. The expression pattern of *cyp19a1b* is similar to estrogen receptors during zebrafish embryo development [32]. It is reported that E2 treatment of *cyp19a1b*-GFP (green fluorescent protein) transgenic embryos results in the appearance of GFP expression in the brain as early as 25 hpf [32,33]. Vitellogenin (VTG, encoded by vtg1/3), the precursor of yolk proteins, is produced by the liver cells of females in response to 17 β-estradiol and is loaded to oocytes [25,34].

To investigate the function of *esr2b* in zebrafish development, we also successfully generated *esr2b* knockout zebrafish lines using TALENS technology. The present study aimed to compare the differences in fertility, embryonic development and sex ratios between *esr2b* knockout and wild-type zebrafish. Furthermore, the expression changes of estrogen receptor downstream response genes *cy19a1b* and *vtg1* were detected using qPCR and in situ hybridization. Our results suggest that *esr2b* is involved in oocyte development and sex differentiation in zebrafish.

## 2. Materials and Methods

### 2.1. Animals and Treatment

The AB-strain zebrafish used in this study were bred and maintained in our laboratory under controlled conditions of a 12 h light:12 h dark cycle at a constant water temperature of 28 °C. The offspring of sexually mature zebrafish were obtained through artificial insemination or natural reproduction. The zebrafish used for in situ hybridization had PTU added, which inhibits pigment production, at a concentration of 0.3 mM when they reached gastrulation stage, and the fish culture water was changed approximately every 24 h. All procedures were conducted in accordance with the Guiding Principles for the Care and Use of Laboratory Animals and were approved by the Special Committee of Science Ethics, Academic Committee of Hunan University of Arts and Sciences (2023090652).

17β-Estradiol (Sigma-aldrich, St. Louis, MO, USA) was dissolved in DMSO and diluted in cultured water to 1 μM. Groups of 30 embryos were kept in Petri dishes (diameter 4 cm) from 2 days post fertilization (dpf) to 4 dpf. Then, samples were collected for RNA extraction or in situ hybridization.

### 2.2. Construction of esr2b Knockout Zebrafish

The Gold gate method was used for the assembly of TALENs elements [35]. The recognition sequences of the left and right arms were AGCTCCTCCCCTG and CCTTGCTGGAGT, respectively. The Fokl backbone plasmids were PCS2-Fokl-KKR and PCS2-Fokl-ELD [36]. The assembled left and right arm plasmids were linearized through NotI digestion and subsequently recovered. Subsequently, the synthesized RNA was transcribed in vitro using an mMESSAGE mMACHINE SP6 Kit (Invitrogen, Carlsbad, CA, USA) and then recovered utilizing an RNeasy Mini Kit (Qiagen, Duesseldorf, Germany). The left and right arm mRNAs were mixed at a final concentration of 100 ng/μL and added to phenol red before microinjection. The P0 embryos that underwent injection were raised until maturity and subsequently crossed with the control zebrafish in order to obtain the F1 knockout zebrafish. The caudal fin tissue of the F1 generation zebrafish was collected to extract the genome to detect the mutation type. Female and male F1 generation zebrafish with the same mutation type were selected for self-crossing to obtain the F2 generation. The genome was extracted from the caudal fin tissue of the F2 generation and the homozygous zebrafish were screened by PCR. The three primers designed to screen homozygotes were *esr2b*-Forward: 5′-GCCCCTGTCCTGGACTCC-3′; *esr2b* KO-Forward:5′-GCAAAAGTCATACTGACCCCAAA; and *esr2b*-Reverse: 5′-GCAAAAGTCA TACTGA CCCCAAA.

### 2.3. Spawning and Fertility

The wild-type zebrafish and *esr2b* knockout homozygous zebrafish were simultaneously introduced into the same system, where they were subjected to identical breeding conditions. The breeding density was 3 fish per liter and breeding was carried out until sexual maturity (3 months); then, the number of male and female individual zebrafish was counted. In order to investigate the impact of *esr2b* knockout on zebrafish reproductive capacity, at least 8 pairs of *esr2b* knockout zebrafish were selected for self-breeding or mating with wild-type zebrafish. Subsequently, the total number of eggs was counted and transferred into Petri dishes. At the gastrula stage, the developmental process of different embryos was recorded (6 h post fertilization (hpf) and 9 hpf). At 24 hpf, embryos were picked out and the number of deaths counted. All experiments were repeated twice each.

### 2.4. RNA Extraction and Quantitative PCR

Zebrafish RNA was extracted using an Ultra-Pure Total RNA Extracting Kit (Simgen, Hangzhou, China). The concentration of RNA was assessed with a NanoDrop 8000 UV-Vis Spectrophotometer (Thermo, Waltham, MA, USA). A cDNA first chain synthesis kit (Simgen, Hangzhou, China) was used for cDNA synthesis. Genomic DNA was eliminated by adding RNase-free DNase. cDNA samples were diluted 5-fold for quantitative PCR (qPCR), according to the manufacture’s advice. qPCR was performed using SYBR^®^ Green Real-time PCR Mater Mix-plus (TOYOBO, Sigaken, Japan) and the Bio-Rad CFX96 System (Bio-Rad, Hercules, CA, USA). The real-time PCR program used was as follows: 95 °C for 2 min, followed by 40 cycles at 95 °C for 15 s, 60 °C for 15 s and 72 °C for 20 s. The primers used for qPCR were as follows: cyp19a1b: 5′-AAAGAGTTACTAATAAAGATCCACCGGTAT-3′ and 5′-TCCACAAGC TTTCCCATTTCA-3′; Vtg1: 5′-ACTACCAACTGGCTGCTTAC-3′ and 5′-ACCATCGGC ACAGATCTTC-3′. *β*-*catin* was used as an internal reference. The *β*-*catin* primer sequences were 5′-CGAGCAGGAGATGGGAACC-3′ and 5′-CAACGGAAACGCTCATTGC-3′. The 2-ΔΔct method was used for measuring the expression levels of *Cyp19a1b* and *Vtg1* [37].

### 2.5. Whole-Mount In Situ Hybridization

Zebrafish embryos at different stages (20 to 30 embryos) were fixed in 4% PFA/PBS solution overnight at 4 °C and subsequently dehydrated with methanol and stored at −20 °C. Whole mount in situ hybridization was performed as reported by Thisse and Thisse (2008) [38]. To prepare the probe, fragments of about 500 bp of zebrafish *cyp19a1b* were cloned and inserted into PCS2+ vector for probe synthesis. The primers are as follows: Forward primer 5′CGGGATCCACAAGGCATCATCTTCAACAGC-3′; Reverse primer 5′GCTCTAGA GCAGCGATCACCATCTCCAGCA-3′. BanHI and XbaI were chosen to generate the recombinant plasmid. The constructed vector was linearized with BamHI and then used as a template to synthesize digoxigenin (DIG)-labeled RNA probes. For details, refer to Song et al., 2021 [39].

### 2.6. Statistics

The results are presented as mean ± standard error of the mean (S-E-M) values. Statistical analyses were performed using SPSS 26.0 (SPSS, Chicago, IL, USA) (*p* < 0.05 was considered statistically significant). All the data were analyzed by Student’s *t*-test or one-way analysis of variance (ANOVA), with multiple comparisons of means performed using Tukey’s test method.

## 3. Results

### 3.1. Generation of esr2b Knockout Zebrafish

**Figure 1:**
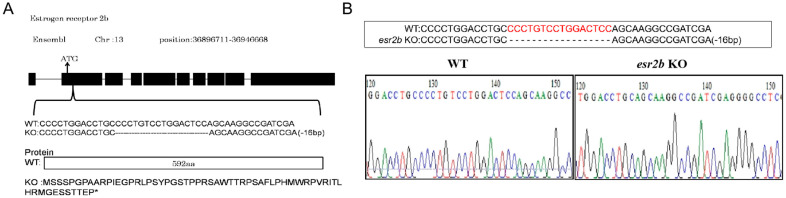
Genomic structure and targeted genetic modification of *esr2b* in zebrafish. (**A**) Gene structure of *esr2b*. Zebrafish *esr2b* contains 10 exons, with the translation initiation codon located on the second exon. Deletion of 16 nucleotides results in translation frameshift and early termination. * represent translation termination. Theoretically, the *esr2b* mRNA of knockout zebrafish only coding 60 amino acids. (**B**) Verified sequence change in *esr2b* knockout zebrafish.

To investigate the function of *esr2b* in reproductive endocrinology, we generated an *esr2b* knockout zebrafish line using TALENS. The *esr2b* gene sequence was downloaded from the Ensembl database and the knockout site was designed in the second exon of the *esr2b* gene, after the initiation codon ATG (Figure 1A). As shown in the sequencing results, we screened homozygous zebrafish with 16 nucleotides deleted (Figure 1B). The translation of the *esr2b* gene was terminated prematurely after the knockout.

### 3.2. Esr2b Knockout Results in Developmental Delay in Zebrafish Embryos

**Figure 2:**
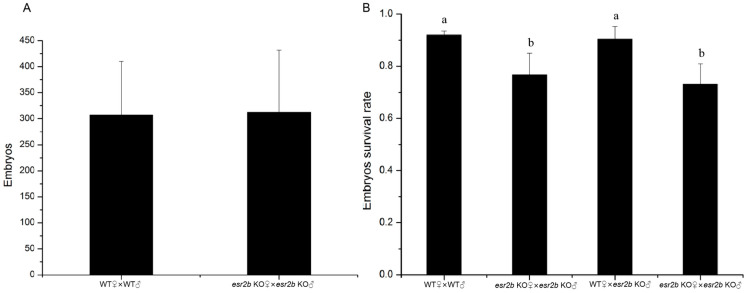
Comparison of fertility and survival rate between wild-type and *esr2b* knockout zebrafish. (**A**) The average number of embryos produced by wild-type and *esr2b* knockout homozygote zebrafish, (**B**) The survival rate of four kinds of embryos (wt♀ × wt♂, *esr2b* KO♀ × *esr2b* KO♂, wt♀ × *esr2b* KO♂, *esr2b* KO♀ × WT♂). Eight pairs from different zebrafish lines (8 male and 8 female) were selected for spawning. The results are presented as mean ± SEM values. Values accompanied by different letters are significantly different (ANOVA followed by Tukey’s test, *p* < 0.05).

**Figure 3:**
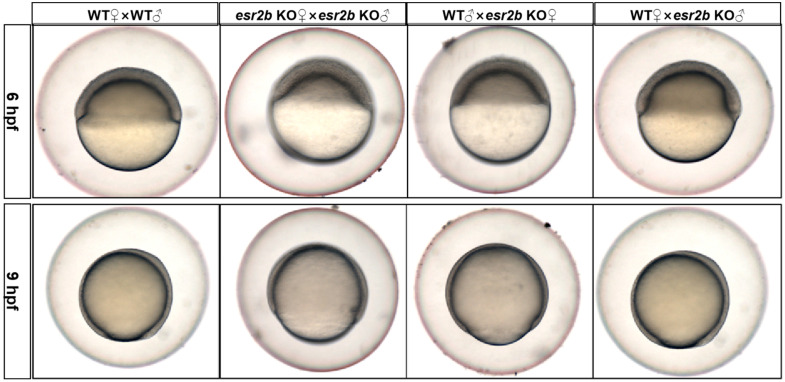
The developmental delay of *esr2b* knockout zebrafish. The developmental situation of zebrafish embryos was observed and photographed at 6 hpf and 9 hpf, respectively. All embryos from different zebrafish lines were collected and randomly transferred into Petri dishes (30/each).

There was no significant difference in the average amount of egg production between *esr2b* knockout zebrafish and wild-type zebrafish (Figure 2A). The survival rate of *esr2b* knockout homozygous embryos at 24 hpf was significantly lower, with only a 76% survival rate compared to the wild-type embryos, which had a survival rate of 92% (Figure 2B). The comparison between the early development of wild-type embryos and *esr2b* knockout embryos revealed a significant delay in the embryonic development of *esr2b* knockout homozygous embryos during the gastrula period (5–10 hpf) (Figure 3). Subsequently, the *esr2b* knockout zebrafish were subjected to reciprocal crosses with the wild-type.The results show that the embryo development speed of hybrid embryos derived from *esr2b* knockout females and wild-type males was comparable to that of *esr2b* knockout homozygous embryos, while the survival rate at 24 h post-fertilization (hpf) was 73% (Figure 2B and Figure 3). Conversely, there was no significant difference in developmental speed between hybrid embryos derived from *esr2b* knockout males mating wild-type females and wild-type zebrafish embryos, with a survival rate of 90% for hybrid embryos at 24 hpf (Figure 2B and Figure 3). 

### 3.3. Esr2b Knockout Results in Male-Biased Sex Ratio

**Figure 4:**
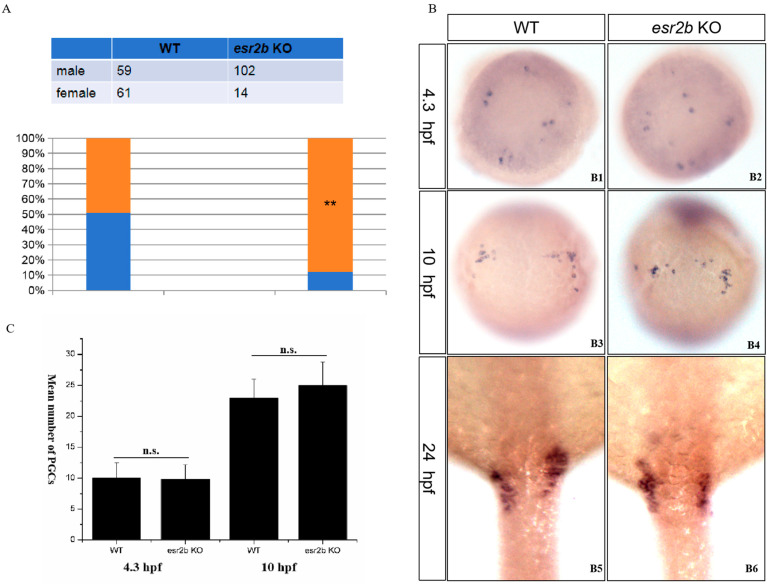
The influence of *esr2b* knockout on sex ratio and the number of PGCs during development. (**A**). Sex ratios of *esr2b* knockout zebrafish and wild-type zebrafish at 90 dpf. Orange and blue represents the proportion of male and female zebrafish in the group, respectively. (**B**) Whole-mount in situ hybridization with *vasa* mRNA probe during early development. At 4.3 hpf, also named the dome stage, the average number of *vasa* signals in wild-type and *esr2b* KO embryos were 10 (*n* = 15) and 9.8 (*n* = 15) (**B1**,**B2**); At 10 hpf, also named the bud stage, the average number of *vasa* signals in wild-type and *esr2b* KO embryos were 22.9 *(n* = 15) and 25 (*n* = 15) (**B3**,**B4**); At 24 hpf, the *vasa* signal intensity showed no significant difference (**B5**,**B6**). (**C**) Comparison of the number of PGCs between wild-type and *esr2b* knockout zebrafish at 4.3 hpf and 10 hpf. n.s.; not significant. **, *p* < 0.01.

Wild-type and *esr2b* knockout zebrafish were cultured to sexual maturity (3 months). The number of male and female individuals in the population were counted. The sex ratio of adult wild-type zebrafish was approximately 1:1, whereas the population of *esr2b* knockout homozygous zebrafish exhibited a significantly higher proportion of males compared to females, with a male-female ratio of about 1:7 (Figure 4A). Whole-mount in situ hybridization was conducted to detect the numbers of PGCs using a *vasa* probe. At 4.3 hpf and 10 hpf, the numbers of PGCs did not appear to be affected in *esr2b* knockout zebrafish (Figure 4(B1)–(B4),C). At 24 hpf, the *vasa* mRNA signal intensity also showed no difference between wild-type and *esr2b* knockout zebrafish (Figure 4(B5),(B6)).

### 3.4. Effects of esr2b Knockout on cyp19a1b Expression and Response to 17β-Estradiol during Development

**Figure 5:**
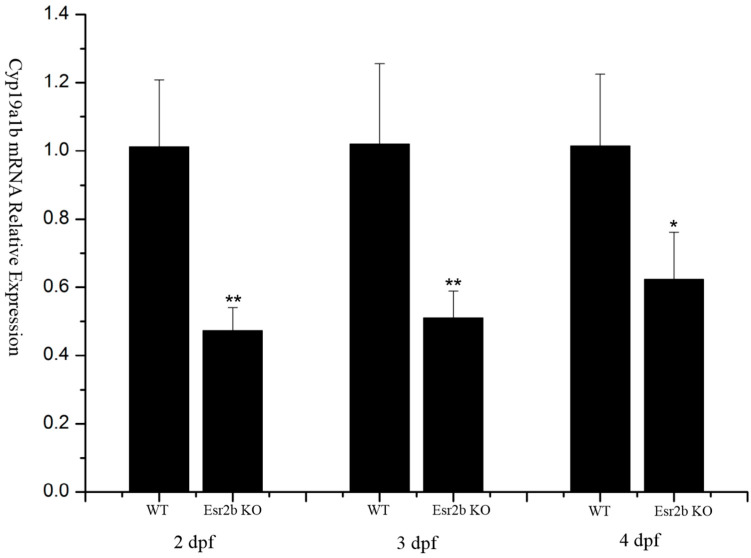
Reduced expression levels of *cyp19a1b* mRNA in *esr2b* knockout zebrafish at 2 dpf, 3 dpf and 4 dpf. (*, *p* < 0.05, **, *p* < 0.01). Group of 30 wild-type or *esr2b* knockout zebrafish embryos were cultured in Petri dishes (4 cm in diameter). At 2 dpf, 3 dpf and 4 dpf, four individual samples of wild-type or *esr2b* knockout zebrafish embryos were collected for RNA extraction. Levels of *cyp19a1b* mRNA relative to *β*-*actin* were detected by qPCR.

**Figure 6:**
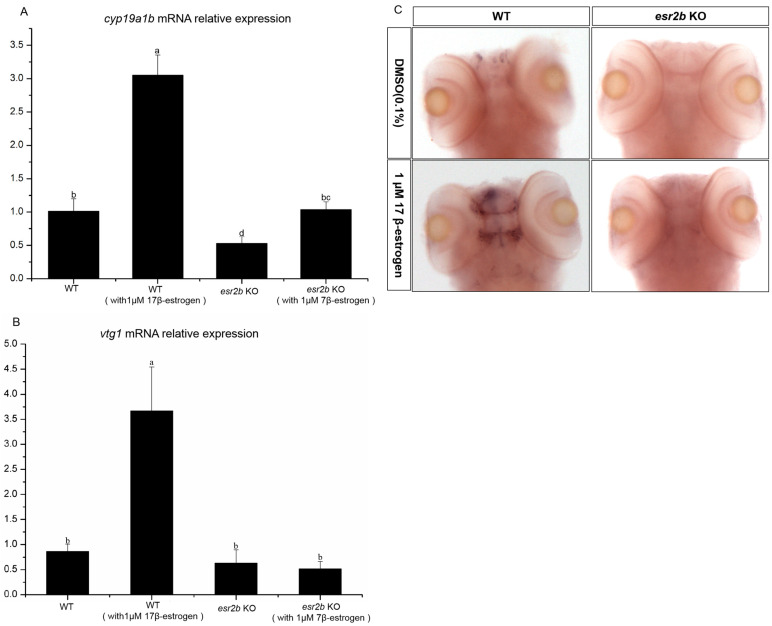
Response of *cyp19a1b* and *vtg1* expression to estrogen stimulation. (**A**,**B**) Groups of 30 zebrafish embryos were treated from 2 to 4 dpf with 1 μM 17β-estradiol (E2). Levels of *cyp19a1b* and *vtg1* mRNA relative to *β*-*actin* RNA were determined by qPCR. The results are presented as mean ± SEM values. Values accompanied by different letters are significantly different (ANOVA followed by Tukey’s test, *p* < 0.05). (**C**) *cyp19a1b* expression analysis by whole-mount in situ hybridization.

*Cyp19a1b* is a well-known estrogen response gene. qPCR was used to detect the effects of *esr2b* knockout on the expression of *cyp19a1b* at different developmental stages. At 2 dpf, 3 dpf and 4 dpf, *cyp19a1b* expression in *esr2b* knockout zebrafish was significantly lower than that in wild-type zebrafish (Figure 5). The treatment of 2 dpf wild-type and *esr2b* knockout zebrafish with exogenous estradiol until 4 dpf resulted in a three-fold increase in the expression of *cyp19a1b* in wild-type zebrafish (Figure 6A). However, in *esr2b* knockout zebrafish, *cyp19a1b* still exhibited responsiveness to estradiol induction—its expression level only showed a 1.9-fold increase (Figure 6A). The results of the in situ hybridization also showed that the *cyp19a1b* mRNA probe signal intensity in *esr2b* knockout zebrafish was significantly weaker than that in wild-type zebrafish at 4 dpf and that the *cyp19a1b* mRNA probe signal intensity in wild-type zebrafish was significantly deepened after estradiol treatment (Figure 6B). Additionally, estradiol treatment significantly stimulated *vtg1* expression in wild-type zebrafish. In contrast, the induction of estradiol on *vtg1* mRNA was disrupted (Figure 6B).

## 4. Discussion

The estrogen receptor signaling pathway plays important roles in vertebrate development and reproduction. Estrogen receptor (ER) gene deletion in mice leads to severe reproductive damage. There are four estrogen receptors found in zebrafish—namely, *gper*, *esr1*, *esr2a* and *esr2b*. In order to understand the role of different estrogen receptors in fish reproduction and development, researchers have knocked out different estrogen receptors by TALENs or CRISPR technology. The egg production of *gper* knockout zebrafish was compared to that of wild-type zebrafish by Wu within a two-week period; the findings revealed that the absence of *gper* caused a significant decrease in female zebrafishes’ egg production, with a reduction of 40.5% [17]. However, other estrogen receptor knockout results showed that *esr1*, *esr2a* and *esr2b* knockout alone did not affect the reproductive ability of female and male zebrafish, and the embryonic development and hatching of their offspring were normal [23]. In this study, a zebrafish line with a 16 bp nucleotide deletion of the *esr2b* gene was successfully constructed using TALENs technology, which caused premature termination of *esr2b* gene translation and failure to produce normal *esr2b* protein. Similar to Lu’s results, we also found that the fertility of *esr2b* knockout zebrafish was not affected, and there was no significant difference in egg production between *esr2b* knockout female zebrafish and wild-type zebrafish. Interestingly, the early development speed of *esr2b* knockout embryos was significantly delayed, and the 24 hpf survival rate of *esr2b* knockout embryos was significantly lower than that of wild-type embryos. The reciprocal cross experiments between *esr2b* knockout zebrafish and wild-type zebrafish demonstrated that the absence of *esr2b* mRNA did not impact the reproductive capacity of male zebrafish, but led to a reduction in the oocyte quality of female zebrafish, ultimately resulting in decreased embryo survival rates at 24 hpf. The follicles’ development is divided into five different stages according to size and morphological criteria [40]. The five stages include Stage I (<140 µm), Stage II (140–340 µm) follicles, Stage III (340–690 µm) and Stage IV (690–730 µm) [40]. It has been reported that *gper* knockout causes a notable decline in the number of oocytes at Stage V [17]. However, the number of Stage III oocytes in the ovaries of *gper* knockout zebrafish is significantly higher compared to that of wild-type zebrafish [17]. *Esr2a* or *esr2b* single knockout does not influence zebrafish oocyte development [23]. Unlike previous *esr2b* knockout results in zebrafish, the present study showed that the early development speed of *esr2b* knockout embryos was significantly delayed and that the 24 hpf survival rate of *esr2b* knockout embryos was significantly lower than that of wild-type embryos. Subsequently, the reciprocal cross experiments demonstrated that the embryo developmental delay and low survival rate were caused by the oocytes of *esr2b* knockout zebrafish. In conclusion, we speculate that *esr2b* is involved in oocyte development, just like *gper*.

The ratio of females to males in the offspring of *esr2b* knockout zebrafish was about 1:7. *Esr1*, *esr2a* and *esr2b* single knockout zebrafish display a male-biased sex ratio (70–85%) [23]. *Esr2a* and *esr2b* double knockout or *ear1*, *esr2a* and *esr2b* triple knockout both cause there to be no female individuals in the group [23]; these results are similar to this study. The mechanism of sex differentiation in fish is complex, not only affected by genes, but also by environmental factors. The mechanisms of sex determination in zebrafish are currently poorly understood and are thought to be polygenic in control [41,42,43,44,45]. However, there is a strong correlation between the number of primordial germ cells (PGCs) and sex differentiation during early zebrafish development. In zebrafish, the deletion of PGC results in the development of more male individuals [46]. In the present study, the results of the in situ hybridization experiments indicated that the number of PGCs before 24 hpf did not show any significant difference between wild-type and *esr2b* knockout embryos. This suggests that the absence of *esr2b* does not influence the male/female ratio in offspring by affecting the quantity of PGCs during embryonic early development.

Aromatase is a key enzyme catalyzing estrogen production. There are two aromatase genes—*cyp19a1a* and *cyp19a1b*—in zebrafish, and *cyp19a1b* is mainly expressed in glial cells in the brain [31,47]. The *cyp19a1b* gene is a classical downstream response gene of the estrogen receptor signaling pathway that can be induced by estrogen, and its promoter sequence contains estrogen response elements [32,48]. Exposure to the estrogen receptor antagonist ICI_182780_ significantly suppresses *cyp19a1b* expression in zebrafish during early developmental stages (24–72 hpf) [32]. *Vtg1* is another downstream response gene of the classical estrogen receptor signaling pathway. It is generally believed that *vtg1* proteins are synthesized in the liver, produced by the liver under the stimulation of estradiol in the blood and then transported to the egg cell [25,26]. To investigate the specific roles of the three different estrogen receptors in regulating downstream affected genes, *esr1*, *esr2a* and *esr2b* MO injection experiments have been performed in zebrafish [49]. The MO study showed that both *esr1* and *esr2b* MO injection blocked the induction of *vtg* by estrogen [49]. Only *esr2b* MO blocked the induction of *cy19a1b* by estrogen [49]. In the present study, *cyp19a1b* expression in *esr2b* knockout zebrafish at different developmental stages (2 dpf–4 dpf) was detected by qPCR. The results showed that *cyp19a1b* mRNA levels in *esr2b* knockout zebrafish were significantly lower than those in wild-type embryos at 2dpf, 3dpf and 4dpf. This suggests that *esr2b* is involved in the regulation of *cy19a1b* gene expression during early developmental stages in zebrafish. However, estrogen treatment experiments showed that *esr2b* knockout only reduced the induction of *cyp19a1b* by estrogen, but did not eliminate the induction effect, suggesting that other estrogen receptors are involved in the regulation of *cy19a1b* expression. In the present study, estrogen exposure assays showed that *esr2b* knockout resulted in the loss of estrogen induction on *vtg1*, suggesting that *esr2b* is involved in the regulation of *vtg1* expression.

## 5. Conclusions

In this study, an *esr2b* knockout zebrafish line with 16 nucleotides deleted was constructed using TALENs technology. *Esr2b* knockout did not affect the fecundity of male and female zebrafish, but affected the quality of female zebrafish oocytes, which was ultimately manifested as delayed embryonic development and a significant increase in mortality during early development. *Esr2b* knockout resulted in a significantly higher number of males than females in the offspring. *Esr2b* knockout also decreased the expression of the estrogen synthesis gene *cyp19a1b* and reduced its ability to respond to estrogen. It also completely disrupted the induction of the responsive gene *vtg1* by estrogen. Our study provides new insights into the role of *esr2b* in zebrafish embryonic development, reproduction and sex differentiation.

## Data Availability

Data can be obtained from the corresponding author upon reasonable request.
